# Effects of Intraoperative Fluid Management on Postoperative Outcomes After Pericardiectomy

**DOI:** 10.3389/fsurg.2021.673466

**Published:** 2021-08-04

**Authors:** Likui Fang, Hong Zheng, Wenfeng Yu, Gang Chen, Fangming Zhong

**Affiliations:** ^1^Department of Thoracic Surgery, Affiliated Hangzhou Chest Hospital, Zhejiang University School of Medicine, Hangzhou, China; ^2^Department of Nursing, Affiliated Hangzhou Chest Hospital, Zhejiang University School of Medicine, Hangzhou, China

**Keywords:** intraoperative fluid management, constrictive pericarditis, pericardiectomy, postoperative complications, enhanced recovery after surgery

## Abstract

**Background:** The effects of intraoperative fluid management on the patients with constrictive pericarditis undergoing pericardiectomy remain unclear. This study explored the relationship between intraoperative fluid management and postoperative outcomes in these patients.

**Methods:** We retrospectively studied 92 patients with constrictive pericarditis undergoing pericardiectomy and assigned them to the restrictive group and the liberal group according to the intraoperative total fluid infusion rate. Postoperative outcomes were compared between the two groups. Binary logistic regression analysis was performed to determine the relationship between the intraoperative total fluid infusion rate and postoperative outcomes.

**Results:** There were 46 (50.0%) cases in the restrictive group and 46 (50.0%) cases in the liberal group. Compared with the liberal group, the restrictive group had significantly lower incidences of postoperative complications and cardiac complications (*P* = 0.005 and *P* = 0.006, respectively). Binary logistics regression analysis also showed the increased risks of postoperative complications (OR, 3.551; 95% CI, 1.192–10.580; *P* = 0.023) and cardiac complications (OR, 6.037; 95% CI, 1.472–25.052; *P* = 0.013) at the liberal group. In addition, the restrictive group had shorter postoperative hospital stay (*P* = 0.026) in comparison to the liberal group.

**Conclusion:** In patients with constrictive pericarditis undergoing pericardiectomy the intraoperative total fluid infusion rate was significantly associated with postoperative outcomes. Restrictive fluid management strategy was related to the positive effects on enhanced recovery after surgery and could be advised as the preferred intraoperative fluid management policy.

## Introduction

Constrictive pericarditis is a rare disease with poor prognosis (1). The inelastic pericardium leads to impaired filling and diastolic dysfunction (2). The etiology of constrictive pericarditis varies widely. In developing countries, tuberculosis is the major cause, while in developed countries the most frequent causes are idiopathic, post-cardiac surgery and post radiation (3, 4). Constrictive pericarditis is chronic and progressive in most cases (4). As a result, conservative treatment is only used as a temporary measure and surgical pericardiectomy is necessary to relieve the pericardial constriction (5–7). However, despite being considered effective, pericardiectomy is accompanied with high incidence of postoperative complications and in-hospital mortality (8–10).

The disordered hemodynamics is a common complication after pericardiectomy and a major cause of in-hospital death (11, 12). The status of hemodynamics is significantly associated with fluid management which exerts great influence in perioperative course. Optimal fluid management plays an important role in the enhanced recovery after surgery (ERAS) and the improvement of postoperative outcomes (13), but the evidence in pericardiectomy is limiting. This study aimed to explore the effect of intraoperative fluid management on postoperative outcomes and find an optimum range of the intraoperative fluid infusion rate in the patients undergoing pericardiectomy for constrictive pericarditis.

## Methods

### Study Population

We retrospectively reviewed the records of the patients diagnosed as constrictive pericarditis in our department between November 2012 and June 2020. The patients were excluded if they were not performed pericardiectomy or if their data of intraoperative fluid infusion were missing. Finally, a total of 92 patients were enrolled. Their perioperative characteristics were extracted from the hospital electronic medical records system. The study protocol was approved by the Institutional Review Board of Affiliated Hangzhou Chest Hospital, Zhejiang University School of Medicine.

The preoperative diagnosis of constrictive pericarditis mainly depended on the clinical symptoms, echocardiography, chest enhanced computed tomography and central venous pressure (CVP). CVP was measured through the catheter placed in internal jugular vein. Pericardiectomy was routinely performed by median sternotomy in all patients without the use of cardiopulmonary bypass. The extent of pericardiectomy included at least the anterolateral pericardium between the two phrenic nerves, the basal pericardium over the diaphragmatic surface, the pericardium on the great arteries and the pericardium from superior vena cava-right atrium junction to inferior vena cava-right atrium junction (14).

### Exposure Variable

The exposure variable was the intraoperative total fluid infusion rate. The volume of intraoperative total fluid was collected from the anesthesia record and defined as the volumes of crystalloid, colloid, and blood products administered between initiation of anesthesia care and arrival in the postanesthesia care unit (15). The crystalloid was Ringer solution and the colloid was hydroxyethyl starch. Hydroxyethyl starch was not used in the patients with renal dysfunction. The intraoperative total fluid infusion rate (ml/kg/h) was defined as the intraoperative total fluid volume per kilogram of weight divided by the operation duration.

### Statistical Analysis

The correlation between the intraoperative total fluid infusion rate and postoperative complications was analyzed by the receiver operating characteristic (ROC) curve and the optimal cutoff value of intraoperative total fluid infusion rate was determined by calculating the Youden Index. The enrolled patients were divided into the restrictive group and the liberal group according to the cutoff value. The measurement data of the two groups were statistically analyzed with the *t*-test. The χ^2^-test, the corrected χ^2^-test or the Fisher exact test was used for the enumeration data, depending on the actual situation. Binary logistic regression analyses were performed to determine the relationship between the intraoperative total fluid infusion rate and postoperative outcomes. Confounders were included, based on univariate analysis. These analyses were conducted using SPSS software (version 24.0, IBM SPSS Inc. United States). Statistical significance was set at *P* < 0.05 (all *P*-values presented were two-sided).

## Results

### Group Division

**Figure 1:**
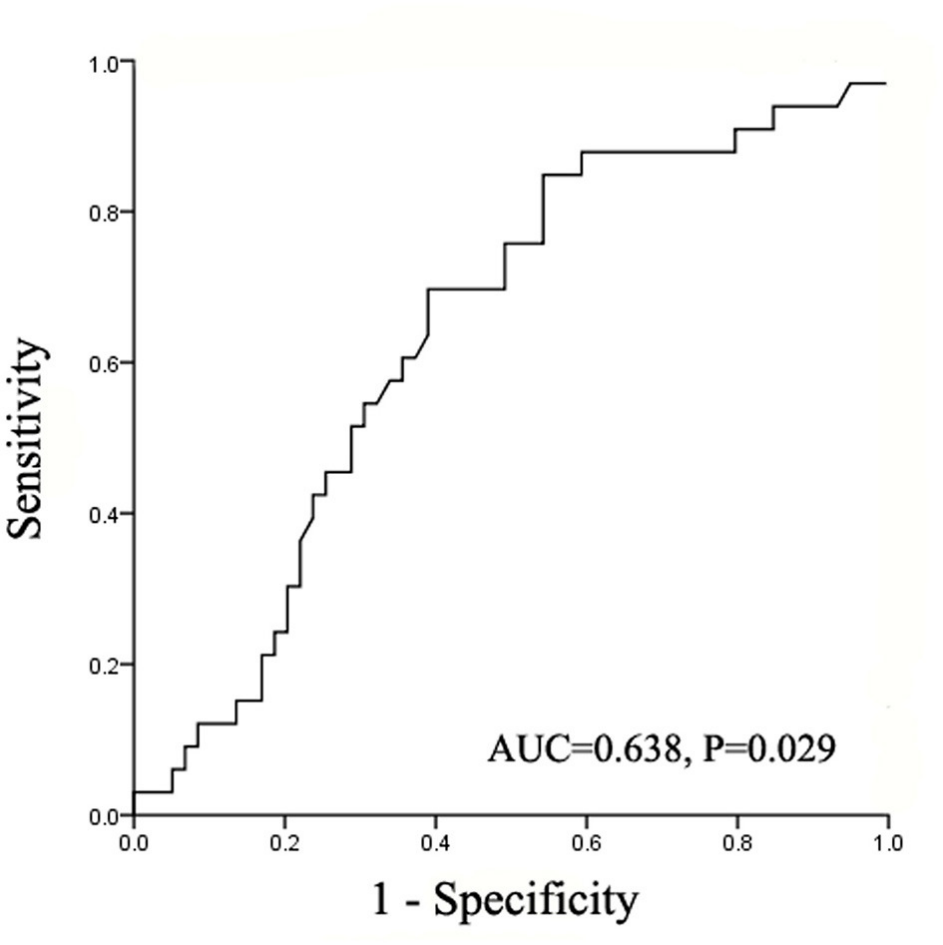
The area under the ROC curve for postoperative complications determined using intraoperative total fluid infusion rate. ROC, receiver operating characteristic; AUC, area under the curve.

The result of ROC curve showed that the intraoperative total fluid infusion rate statistically correlated with postoperative complications. The area under curve (AUC) was 0.638 (95% CI = 0.521–0.755, *P* = 0.029) (Figure 1). The Youden Index was further calculated and the result showed the optimal cutoff value of the intraoperative total fluid infusion rate was 7.47 ml/kg/h (sensitivity 69.7%, specificity 61.0%, Youden Index 0.307). According to the cutoff value, the patients were divided into the restrictive group and the liberal group. In the restrictive group, the range of intraoperative total fluid infusion rate was from 2.68 to 7.46 ml/kg/h (median = 5.99). In the liberal group, the range was from 7.47 to 20.55 ml/kg/h (median = 9.45).

### Baseline Characteristics

A total of 92 patients were enrolled in this study, with 46 (50%) cases in the restrictive group and 46 (50%) cases in the liberal group. Table 1 presented the comparative results of baseline characteristics between the two groups.

**Table 1:** Clinical characteristics of the patients based on the intraoperative total fluid infusion rate.

**Variables**	**Restrictive group (*N* = 46)**	**Liberal** ** group** ** (*N* = 46)**	***P*-value**
Gender			0.625
Male	36 (78.3%)	34 (73.9%)	
Female	10 (21.7%)	12 (26.1%)	
Age, years	56 (16–80)	61 (17–83)	0.235
Etiology			1.000
Tuberculosis	43 (93.5%)	42 (91.3%)	
Other	3 (6.5%)	4 (8.7%)	
Symptom duration, months	2.0 (0.3–18.0)	2.0 (0.3–12.0)	0.140
Preoperative NYHA functional class			0.258
I	4 (8.7%)	3 (6.5%)	
II	19 (41.3%)	12 (26.1%)	
III	22 (47.8%)	27 (58.7%)	
IV	1 (2.2%)	4 (8.7%)	
Hypertension	6 (13.0%)	9 (19.6%)	0.397
Diabetes	3 (6.5%)	2 (4.3%)	1.000
Atrial fibrillation	5 (10.9%)	8 (17.4%)	0.369
BMI, kg/m^2^	21.7 (18.0–28.7)	20.0 (16.3–27.5)	0.003
SBP, mmHg	117 (90–147)	115 (91–156)	0.645
DBP, mmHg	80 (50–114)	78 (62–106)	0.227
Pulse rate (beats/min)	105 (67–145)	98 (76–145)	0.399
Preoperative CVP, cmH_2_O	28.5 (18.0–42.0)	26.0 (15.5–35.0)	0.029
Pleural effusion	43 (93.5%)	44 (95.7%)	1.000
Ascites	25 (54.3%)	24 (52.2%)	0.834
Pericardial effusion	38 (82.6%)	35 (76.1%)	0.440
LVEF, %	58.1 (39.9–78.0)	58.0 (46.0–72.0)	0.284
Hemoglobin, g/dl	124 (94–167)	122 (90–151)	0.239
Albumin, g/L	33.1 (25.1–48.8)	32.1 (24.7–45.2)	0.095
Total bilirubin, μmol/L	18.1 (6.4–66.7)	16.6 (4.4–59.2)	0.131
Direct bilirubin, μmol/L	10.8 (3.2–42.5)	9.1 (2.7–50.3)	0.261
Serum sodium, mmol/L	138.2 (126.7–144.5)	137.2 (129.5–143.1)	0.750
Serum potassium, mmol/L	4.0 (2.8–5.1)	3.8 (2.7–5.0)	0.881
Preoperative lactate, mmol/L	1.5 (0.6–2.7)	1.4 (0.5–2.4)	0.865
Preoperative BNP, pg/ml	181 (21–786)	169 (21–961)	0.709
Operative duration, min	270 (140–400)	214 (144–390)	<0.001
Blood loss, ml	125 (40–400)	175 (50–800)	0.154
Intraoperative urine output, mL	650 (50–2,000)	700 (100–1,500)	0.624
Intraoperative crystalloid infusion rate, mL/kg/h	4.9 (2.0–7.5)	7.0 (3.9–17.0)	<0.001
Intraoperative colloid infusion rate, mL/kg/h	1.1 (0–2.9)	2.5 (0–6.9)	<0.001
Intraoperative blood transfusion, ml	0	0	/

*Values presented as N (percentage) for categorical variables and median (range) for continuous variables*.

*NYHA, New York Heart Association; BMI, body mass index; SBP, systolic blood pressure; DBP, diastolic blood pressure; CVP, central venous pressure; LVEF, left ventricular ejection fraction (measured on echocardiogram); BNP, brain natriuretic peptide*.

### Postoperative Outcomes

The comparison of outcomes between the restrictive group and the liberal group was shown in the Table 2. Compared with the liberal group, the restrictive group had significantly lower incidences of postoperative complications and cardiac complications (*P* = 0.005 and *P* = 0.006, respectively). Cardiac complications included 6 cases of low cardiac output, 3 cases of cardiac failure and 4 cased of arrhythmia in the liberal group and 3 cases of low cardiac output in the restrictive group. The incidences of pulmonary complications and acute liver or kidney injury were comparable in the two groups. In addition, the restrictive group had shorter postoperative hospital stay (*P* = 0.026) in comparison to the liberal group. There was no mortality within 30 days after pericardiectomy in the two groups.

**Table 2:** Comparison of restrictive and liberal groups on postoperative outcomes.

**Variables**	**Restrictive group (*N* = 46)**	**Liberal group (*N* = 46)**	***P*-value**
Postoperative lactate, mmol/L	1.9 (0.8–4.8)	1.7 (0.7–4.6)	0.371
Postoperative CVP, cmH_2_O	16.0 (2.0–28.0)	14.0 (5.0–32.0)	0.757
Postoperative BNP, pg/ml	208 (41–1,553)	188 (27–1,515)	0.808
Postoperative ICU stay, days	2 (0–6)	3 (0–10)	0.178
Postoperative intubation, h	22.5 (0–122.0)	23.0 (0–232.2)	0.167
Duration of using vasoactive agents, h	0 (0–95.0)	1.0 (0–144.0)	0.113
Postoperative complications	10 (21.7%)	23 (50.0%)	0.005
Cardiac complications	3 (6.5%)	13 (28.3%)	0.006
Pulmonary complications	3 (6.5%)	7 (15.2%)	0.180
Acute liver or kidney injury	3 (6.5%)	1 (2.2%)	0.609
Duration of chest drainage, days	12 (5–27)	12 (4–32)	0.853
Postoperative hospital stay, days	14 (9–34)	18 (7–40)	0.026
30-day mortality	0 (0.0%)	0 (0.0%)	/

*Values presented as median (range) for continuous variables and N (percentage) for categorical variables*.

*CVP, central venous pressure; BNP, brain natriuretic peptide; ICU, intensive care unit*.

### Multivariate Analysis

In order to determine the degree of contribution of the intraoperative total fluid infusion rate on postoperative outcomes and cardiac complications, we performed univariate analysis at first and then included the statistically significant factors in multivariate regression model (Supplementary Tables 1, 2). Because nearly one third of the patients had only crystalloid during the surgical procedure, the infusion rate of intraoperative total fluids in these patients was the intraoperative crystalloid infusion rate, so this was excluded in the regression model.

Binary logistic regression analysis demonstrated that compared with the restrictive group, the risk for postoperative complications was significantly increased in the liberal group (OR, 3.551; 95% CI, 1.192–10.580; *P* = 0.023) (Table 3). Similarly, the risk for cardiac complications was also increased in the liberal group (OR, 6.037; 95% CI, 1.472–25.052; *P* = 0.013) (Table 3).

**Table 3:** Effect of intraoperative total fluid infusion rate on postoperative outcomes.

**Groups**	**Postoperative complications**			**Cardiac complications**		
	**OR**	**95% CI**	***P*-value**	**OR**	**95% CI**	***P*-value**
Restrictive group	1	/	/	1	/	/
Liberal group	3.551	1.192–10.580	0.023	6.073	1.472–25.052	0.013

*OR, odds ratio; CI, confidence interval*.

## Discussion

The elevated burden of mortality and morbidity after cardiac surgery presents a tremendous opportunity for enhanced recovery (16). Although ERAS is relatively new to cardiac surgery, the evidence-based protocols have shown promise (17). The infusion volume and rate of intraoperative fluid are important components of ERAS, but clinically applicable standards in pericardiectomy have yet to emerge, and anesthesiologists offer a relatively random adjustment during the surgery, especially in the emergency condition such as hypotension or bleeding. This study provided some references to clinical intraoperative fluid management in the patients undergoing pericardiectomy for constrictive pericarditis. The results of our study supported the view that the difference in infusion rate of intraoperative total fluid was associated with significant differences in postoperative outcomes.

In constrictive pericarditis, heart diastolic function is limited due to the thicken and inelastic pericardium, leading to the hemodynamic paradox of low preload but high filling pressures (18). One of the major complications is the disordered hemodynamics which can be caused by the disease itself and surgical pericardiectomy (19). Because of the myocardial atrophy, acute overdistension of ventricles after dissecting pericardium could lead to cardiac failure, especially in cases of long-standing constriction (3, 20). Additionally, in order to prevent pulmonary edema, the ideal approach is to decorticate the left ventricle before the right ventricle but this is not always technically feasible (21). In theory, optimal intraoperative fluid management could reduce the risks of acute volume overload, ventricular failure, and pulmonary edema.

Extensive comparative studies have been reported in the literature on the intraoperative fluid management in elective non-cardiac surgery. A systematic review and meta-analysis suggested that compared with the liberal fluid policy, the restrictive policy could reduce 35% risk of postoperative complications in elective surgery (22). In addition, a recent large observational study conducted by Shin and his colleagues showed that liberal intraoperative fluid management was associated with increased postoperative complications, length of stay and total cost of hospitalization (23). Intraoperative fluid management should maintain the patient in a euvolaemic state and excessive fluid has been proven to be associated with more harm in non-cardiac surgery (24). Similarly, our study demonstrated that restrictive fluid infusion rate (2.68–7.46 ml/kg/h) was associated with a lower incidence of postoperative complications and cardiac complications but was not linked to an increased risk of acute liver or kidney injury. Postoperative length of stay was significantly increased in the patients receiving liberal fluid infusion rate, presumably because of the need to treat complications. The incidence of postoperative pulmonary complications was also lower in the restrictive group, but the difference was not statistically significant. Numerous studies have suggested that the excessive fluid administration was associated with postoperative pulmonary edema, but it was not the sole culprit and was just one of the anesthetic- and surgery-related risk factors (25).

This study verified the correlation between postoperative outcomes and intraoperative total fluid infusion rate but there are some limitations. First, because this is a single-center retrospective study, selection bias is inevitable. Although the baseline characteristics were comparable between the two groups, it was difficult to completely balance the preoperative conditions of the patients. Second, body mass index and preoperative CVP between groups were uneven, which was related to the grouping variable of fluid infusion rate. Finally, because only a small fraction of patients received colloid, we were not adequately powered to independently reproduce the association between colloid infusion rate and outcomes.

## Conclusion

Although the intraoperative period is a relatively brief portion of the perioperative course, it represents a uniquely vulnerable and complex physiologic state. Intraoperative fluid management strategy could significantly influence postoperative outcomes and should routinely be discussed preoperatively in consultation with anesthesiologists. This study confirmed the difference in infusion rate of intraoperative total fluid was associated with significant differences in postoperative outcomes. Restrictive fluid management strategy was related to the positive effects on ERAS and could be advised as the preferred intraoperative fluid management policy.

## Data Availability Statement

The raw data supporting the conclusions of this article will be made available by the authors, without undue reservation.

## Author Contributions

LF and FZ contributed to the conception, design of the work, and revision of the manuscript. LF and HZ contributed to data analysis and editing the manuscript. WY and GC contributed to data acquisition, statistical analysis, and interpretation of the data. All authors have approved the final draft of the manuscript.

## Conflict of Interest

The authors declare that the research was conducted in the absence of any commercial or financial relationships that could be construed as a potential conflict of interest.

## Publisher's Note

All claims expressed in this article are solely those of the authors and do not necessarily represent those of their affiliated organizations, or those of the publisher, the editors and the reviewers. Any product that may be evaluated in this article, or claim that may be made by its manufacturer, is not guaranteed or endorsed by the publisher.
