# Supplementary material for: Effects of Intraoperative Fluid Management on Postoperative Outcomes After Pericardiectomy
Source: Front Surg. 2021 Aug 4;8:673466. doi: 10.3389/fsurg.2021.673466 (PMC8372555; doi:10.3389/fsurg.2021.673466)
Supplement: Supplementary file 1 [file Data_Sheet_1.docx]

Supplemental table 1. The analysis of perioperative characteristics predicting postoperative complications

| Variables | Postoperative complications | | P value | Multivariate analysis | | |
| --- | --- | --- | --- | --- | --- | --- |
|  | No (N=59) | Yes (N=33) |  | OR | 95%CI | P value |
| Sex  Male  Female | 45 (76.3%)  14 (23.7%) | 25 (75.8%)  8 (24.2%) | 0.956 |  |  |  |
| Age, years | 56 (16-79) | 62 (17-83) | 0.084 |  |  |  |
| Etiology |  |  | 0.099 |  |  |  |
| Tuberculosis | 52 (88.1%) | 33 (100.0%) |  |  |  |  |
| Other | 7 (11.9%) | 0 (0.0%) |  |  |  |  |
| Symptom duration, months | 2 (0.3-18.0) | 2 (0.3-12.0) | 0.057 |  |  |  |
| Preoperative NYHA functional class |  |  | 0.057 |  |  |  |
| Ⅰ | 6 (10.2%) | 1 (3.0%) |  |  |  |  |
| Ⅱ | 23 (39.0%) | 8 (24.2%) |  |  |  |  |
| Ⅲ | 29 (49.2%) | 20 (60.6%) |  |  |  |  |
| Ⅳ | 1 (1.7%) | 4 (12.1%) |  |  |  |  |
| Hypertension | 9 (15.3%) | 6 (18.2%) | 0.715 |  |  |  |
| Diabetes | 4 (6.8%) | 1 (3.0%) | 0.778 |  |  |  |
| Atrial fibrillation | 8 (13.6%) | 5 (15.2%) | 1.000 |  |  |  |
| BMI, kg/m2 | 21.4 (16.7-28.7) | 20.6 (16.3-27.7) | 0.261 |  |  |  |
| SBP, mmHg | 119 (90-156) | 111 (91-146) | 0.180 |  |  |  |
| DBP, mmHg | 78 (60-114) | 79 (50-101) | 0.427 |  |  |  |
| Pulse rate (beats/min) | 100 (67-145) | 107 (80-145) | 0.079 |  |  |  |
| Preoperative CVP, cmH_2_O | 26.0 (16.7-42.0) | 28.0 (15.5-35.0) | 0.539 |  |  |  |
| Pleural effusion | 54 (91.5%) | 33 (100.0%) | 0.215 |  |  |  |
| Ascites | 33 (55.9%) | 16 (48.5%) | 0.492 |  |  |  |
| Pericardial effusion | 48 (81.4%) | 25 (75.8%) | 0.525 |  |  |  |
| Pericardial calcification | 12 (20.3%) | 8 (24.2%) | 0.663 |  |  |  |
| LVEF, % | 59.0 (39.9-78.0) | 57.4 (49.2-73.0) | 0.751 |  |  |  |
| Hemoglobin, g/dl | 124.0 (94.0-167.0) | 122.0 (90.0-151.0) | 0.969 |  |  |  |
| Albumin, g/L | 33.3 (25.1-48.8) | 32.0 (24.7-38.1) | 0.033 | 1.051 | 0.936-1.180 | 0.400 |
| Total bilirubin, μmol/L | 17.1 (4.4-55.6) | 16.7 (6.4-66.7) | 0.683 |  |  |  |
| Direct bilirubin, μmol/L | 10.2 (2.7-42.5) | 9.9 (3.2-50.3) | 0.532 |  |  |  |
| Serum sodium, mmol/L | 138.4 (126.7-144.5) | 136.7 (129.5-143.1) | 0.028 | 1.135 | 0.968-1.330 | 0.119 |
| Serum potassium, mmol/L | 3.9 (2.8-5.1) | 3.9 (2.7-5.0) | 0.704 |  |  |  |
| Preoperative lactate, mmol/L | 1.4 (0.5-2.6) | 1.5 (0.7-2.7) | 0.081 |  |  |  |
| Preoperative BNP, pg/ml | 163 (21-786) | 181 (21-961) | 0.278 |  |  |  |
| Operative duration, min | 243 (140-400) | 250 (157-390) | 0.371 |  |  |  |
| Blood loss, ml | 150 (50-400) | 200 (40-800) | 0.282 |  |  |  |
| Intraoperative urine output, mL | 700 (50-2000) | 650 (50-1800) | 0.496 |  |  |  |
| Infusion rate of intraoperative total fluids |  |  | 0.005 |  |  |  |
| Restrictive | 36 (61.0%) | 10 (30.3%) |  | 1 | / | / |
| Liberal | 23 (39.0%) | 23 (69.7%) |  | 3.551 | 1.192-10.580 | 0.023 |
| Infusion rate of intraoperative colloid, mL/kg/h | 1.6 (0-6.9) | 2.1 (0-4.6) | 0.034 | 1.006 | 0.680-1.488 | 0.975 |
| Postoperative lactate, mmol/L | 1.8 (0.7-4.8) | 1.8 (0.9-4.6) | 0.732 |  |  |  |
| Postoperative CVP, cmH_2_O | 14.0 (2.0-28.0) | 16.5 (4.0-32.0) | 0.070 |  |  |  |
| Postoperative BNP, pg/ml | 225 (27-1553) | 164 (51-1515) | 0.927 |  |  |  |

Values presented as N (percentage) for categorical variables and median (range) for continuous variables.

OR, odds ratio; CI, confidence interval; NYHA, New York Heart Association; BMI, body mass index; SBP, systolic blood pressure; DBP, diastolic blood pressure; CVP, central venous pressure; LVEF, left ventricular ejection fraction (measured on echocardiogram); BNP, brain natriuretic peptide

Supplemental table 2. The analysis of perioperative characteristics predicting cardiac complications

| Variables | Postoperative complications | | P value | Multivariate analysis | | |
| --- | --- | --- | --- | --- | --- | --- |
|  | No (N=76) | Yes (N=16) |  | OR | 95%CI | P value |
| Sex  Male  Female | 56 (73.7%)  20 (26.3%) | 14 (87.5%)  2 (12.5%) | 0.392 |  |  |  |
| Age, years | 57.5 (16-79) | 67.5 (17-83) | 0.130 |  |  |  |
| Etiology |  |  | 0.457 |  |  |  |
| Tuberculosis | 69 (90.8%) | 16 (100.0%) |  |  |  |  |
| Other | 7 (9.2%) | 0 (0.0%) |  |  |  |  |
| Symptom duration, months | 2 (0.3-18.0) | 2 (0.3-12.0) | 0.688 |  |  |  |
| Preoperative NYHA functional class |  |  | 0.053 |  |  |  |
| Ⅰ | 7 (9.2%) | 0 (0.0%) |  |  |  |  |
| Ⅱ | 27 (35.5%) | 4 (25.0%) |  |  |  |  |
| Ⅲ | 40 (52.6%) | 9 (56.3%) |  |  |  |  |
| Ⅳ | 2 (2.6%) | 3 (18.8%) |  |  |  |  |
| Hypertension | 11 (14.5%) | 4 (25.0%) | 0.507 |  |  |  |
| Diabetes | 4 (5.3%) | 1 (6.3%) | 1.000 |  |  |  |
| Atrial fibrillation | 8 (10.5%) | 5 (31.3%) | 0.077 |  |  |  |
| BMI, kg/m2 | 21.2 (16.7-28.7) | 19.7 (16.3-27.5) | 0.208 |  |  |  |
| SBP, mmHg | 116 (90-156) | 113 (94-139) | 0.749 |  |  |  |
| DBP, mmHg | 79 (50-114) | 80 (62-95) | 0.423 |  |  |  |
| Pulse rate (beats/min) | 101 (67-145) | 112 (80-145) | 0.090 |  |  |  |
| Preoperative CVP, cmH_2_O | 27.0 (16.7-42.0) | 29.0 (15.5-35.0) | 0.701 |  |  |  |
| Pleural effusion | 71 (93.4%) | 16 (100.0%) | 0.160 |  |  |  |
| Ascites | 40 (52.6%) | 9 (56.3%) | 0.792 |  |  |  |
| Pericardial effusion | 62 (81.6%) | 11 (68.8%) | 0.417 |  |  |  |
| LVEF, % | 58.0 (39.9-78.0) | 56.0 (49.2-73.0) | 0.869 |  |  |  |
| Hemoglobin, g/dl | 124.0 (94.0-167.0) | 120.0 (90.0-151.0) | 0.581 |  |  |  |
| Albumin, g/L | 33.0 (24.7-48.8) | 31.7 (24.8-37.5) | 0.063 |  |  |  |
| Total bilirubin, μmol/L | 16.7 (4.4-66.7) | 18.6 (9.5-47.9) | 0.720 |  |  |  |
| Direct bilirubin, μmol/L | 10.1 (2.7-50.3) | 12.9 (4.3-33.9) | 0.724 |  |  |  |
| Serum sodium, mmol/L | 138.4 (126.7-144.5) | 136.4 (131.9-142.4) | 0.075 |  |  |  |
| Serum potassium, mmol/L | 3.9 (2.7-5.1) | 3.9 (3.1-5.0) | 0.482 |  |  |  |
| Preoperative lactate, mmol/L | 1.5 (0.5-2.7) | 1.4 (0.8-2.4) | 0.985 |  |  |  |
| Preoperative BNP, pg/ml | 172 (21-786) | 195 (70-961) | 0.197 |  |  |  |
| Operative duration, min | 245 (140-400) | 223 (157-375) | 0.954 |  |  |  |
| Blood loss, ml | 150 (40-800) | 150 (50-400) | 0.467 |  |  |  |
| Intraoperative urine output, mL | 700 (50-2000) | 600 (100-1000) | 0.501 |  |  |  |
| Infusion rate of intraoperative total fluids |  |  | 0.006 |  |  |  |
| Restrictive | 43 (56.6%) | 3 (18.8%) |  | 1 | / | / |
| Liberal | 33 (43.4%) | 13 (81.3%) |  | 6.073 | 1.472-25.052 | 0.013 |
| Infusion rate of intraoperative colloid, mL/kg/h | 1.7 (0-6.9) | 2.0 (0-4.6) | 0.113 |  |  |  |
| Postoperative lactate, mmol/L | 1.8 (0.7-4.8) | 1.7 (0.9-4.6) | 0.758 |  |  |  |
| Postoperative CVP, cmH_2_O | 14.0 (2.0-28.0) | 16.5 (10.0-32.0) | 0.031 | 1.127 | 1.004-1.264 | 0.042 |
| Postoperative BNP, pg/ml | 217 (27-1553) | 148 (57-1258) | 0.927 |  |  |  |

Values presented as N (percentage) for categorical variables and median (range) for continuous variables.

OR, odds ratio; CI, confidence interval; NYHA, New York Heart Association; BMI, body mass index; SBP, systolic blood pressure; DBP, diastolic blood pressure; CVP, central venous pressure; LVEF, left ventricular ejection fraction (measured on echocardiogram); BNP, brain natriuretic peptide
